# A demonstration of using formal consensus methods within guideline development; a case study

**DOI:** 10.1186/s12874-021-01267-0

**Published:** 2021-04-17

**Authors:** P. Carter, K. J. M. O’Donoghue, K. Dworzynski, L. O’Shea, V. Roberts, T. Reeves, A. Bastounis, M. A. Mugglestone, J. Fawke, S. Pilling

**Affiliations:** 1 Centre for Outcomes Research and Effectiveness, Research Department of Clinical, Educational & Health Psychology, University College London, 1-19 Torrington Place, London, WC1E 7HB UK; 2 National Guideline Alliance, Royal College of Obstetricians and Gynaecologists, 10-18 Union Street, London, SE1 1SZ UK; 3 Division of Epidemiology & Public Health, School of Medicine, University of Nottingham, City Hospital, Nottingham, NG5 1PB UK; 4 Leicester Neonatal Service, University Hospitals Leicester Infirmary Square, Leicester, LE1 5WW UK; 5 Camden and Islington NHS Foundation Trust, St Pancras, Hospital, 4 St Pancras Way, London, NW1 0PE UK

**Keywords:** Formal consensus, Guideline development, Methodology

## Abstract

**Background:**

Recommendations within guidelines are developed by synthesising the best available evidence; when limited evidence is identified recommendations are generally based on informal consensus. However, there are potential biases in group decision making, and formal consensus methods may help reduce these.

**Methods:**

We conducted a case study using formal consensus, to develop one set of recommendations within the Neonatal Parenteral Nutrition guideline being produced for the National Institute for Health and Care Excellence. Statements were generated through identification of published guidelines on several topics relating to neonatal parenteral nutrition. Ten high quality guidelines were included, and 28 statements were generated; these statements were rated by the committee via two rounds of voting. The statements which resulted in agreement were then used to develop the recommendations.

**Results:**

The approach was systematic and provided transparency. Additionally, a number of lessons were learnt; including the value of selecting the appropriate topic, giving adequate time to the process, and ensuring methodologies are understood by the committee for their value and relevance.

**Conclusion:**

Formal consensus is a valuable option for use within guideline development when specific criteria are met. The approach provides transparent methodology, ensuring clarity on how recommendations are developed.

**Supplementary Information:**

The online version contains supplementary material available at 10.1186/s12874-021-01267-0.

## Background

Guidelines are developed by the National Institute for Health and Care Excellence (NICE) to improve health and social care. The guidelines are based on the best available evidence and are produced following a detailed manual [1]. The manual focuses on carrying out systematic reviews, anticipating sufficient evidence is available to collate data. Although formal consensus is stated as an option in the NICE manual [1]; if a review is conducted and no evidence is identified, recommendations are commonly made using informal consensus by the committee. The committee discuss their own current practices, and what they believe to be best clinical practice. However, there is no specific structure to the debate.

The effectiveness of group decision making using informal consensus may be open to bias [2]. Despite the NICE guideline processes emphasising that all committee members have equal status, evidence suggests in some situations some individuals may not be as confident in giving their views as openly as others. In contrast other more confident people may dominate discussion [3, 4]. As such, where differences in clinical practice occur, bias may be towards taking on practices which favour the more confident speakers within a group, potentially threatening transparency. There is often a perceived hierarchy within groups, and this may be particularly relevant within a healthcare setting where levels of specialism are accepted [5]. Additionally evidence suggests that often people prefer to provide shared opinions, supporting provided arguments [5, 6]. Or people may not feel able to disagree with long standing accepted opinions on specific topics [7]. It is therefore important to consider these factors, as they may lead to potential biases. Formal consensus methods were developed to overcome these potential biases, giving equal participation to all members of the group, and to provide a transparent and systematic approach to group decision making [8]. Although an option for developing recommendations, and examples such as the ‘Early and Locally advanced breast cancer’ guideline, which used formal consensus methods to develop recommendations on adjuvant systemic therapy, exist, [9] use of formal consensus methods is not routine. Therefore, understanding the practicalities of conducting a formal consensus exercise should be explored, and importantly the methods should be documented and discussed.

This case study relates specifically to the NICE guideline development process; however, it should be noted that the majority of guidelines, across countries and across different clinical conditions are developed following similar methodology [10–12]. For example, the World Health Organization have a ‘*Handbook for Guideline Development’*, which explicitly describes the process of formal evidence synthesis using systematic literature reviews, but does not give guidance on informal or formal consensus methods [11].

### Objective

To conduct a formal consensus exercise as part of guideline development; to determine feasibility and practicalities of carrying out formal consensus where evidence is limited, and a standard systematic literature review is not feasible.

We conducted a case study to demonstrate an instance where a principal, in this case, ‘formal consensus’ has been used. We used a real world situation, conducting the case study as part of a guideline which was in development by the National Guideline Alliance (NGA). We aimed to contextualise the process of conducting a formal consenting procedure within this setting, and therefore decided a case study was the most appropriated methodology for this research [13].

We selected to carry out formal consensus using a formal consenting procedure developed according to aspects of the nominal group technique and the Delphi method, both commonly used in healthcare [8]. The nominal group technique is assumed to be effective in quickly obtaining consensus from a range of participants [14] and the Delphi method is considered a reliable way to gain consensus opinion from a group of experts [15].

## Methods

### Research approach

This case study was conducted as part of the development of the Neonatal Parenteral Nutrition guideline [16]. During the initial phase of the guideline, stakeholders, who included a range of public and private parties with an interest in neonatal parenteral nutrition (NPN), identified certain topics that were considered vital for inclusion (provision of vitamins, electrolytes, minerals [specifically magnesium], trace elements, delivery of lipids via syringe or bags, filtration and protection from light, and fluid volume) [1]. However, it was agreed that it would be difficult to review these topics using the systematic reviewing process because administration would be guided by physiological, pathophysiological and clinical principles and definitive published evidence was unlikely to be available. It would also be unethical to conduct studies withholding certain components of parenteral nutrition. In view of these considerations, it was agreed that formal consensus would be conducted, enabling the committee to make recommendations that address some general principles of NPN, which were needed to provide important guidance.

### Review protocol

A review protocol was developed to ensure a systematic and transparent process was conducted; the protocol and full details of methods were published as part of the guideline [16]. In order to develop the statements for use within the formal consensus exercise we searched for international, national and regional guidelines or clinical protocols on NPN published or updated in the preceding ten years (January 2008 onwards). We excluded local protocols based on the assumption these would be based on either regional, national or international protocols and may not be generalisable. The population included babies born preterm, up to 28 days after their due birth date and babies born at term, up to 28 days after their birth.

### Search strategy

Search strategies were developed (see Additional file 1.) and included medical subject headings and free text terms based on the review protocol eligibility criteria, including a filter for guidelines. The search was conducted on Ovid Medline (R) In-process & Other Non-Indexed Citations and Ovid MedLINE(R) (1946 onwards), Embase and Embase Classic (1947 onwards) to 11th December 2018. A general web search using free text terms based on the inclusion criteria was also conducted to locate any relevant guidelines not indexed on the Medline and Embase databases.

### Study selection

Retrieved titles and abstracts were imported into an in-house database. Initial screening of titles and abstracts was conducted by one reviewer; a 10 % random sample was then screened by a second reviewer, any discrepancies were resolved by discussion. Full texts of potentially relevant articles were obtained and independently screened by two reviewers.

### Quality assessment

Potentially relevant guidelines were independently assessed for quality by two reviewers using the Appraisal of Guidelines for Research and Evaluation (AGREE II) instrument [17]. The tool assesses six domains: scope and purpose, stakeholder involvement, rigour of development, clarity of presentation, applicability and editorial independence. Within each domain there is a set of questions, each of which is scored using a 7-point Likert scale (1 – ‘strongly disagree’ to 7 – ‘strongly agree’), and an overall score is calculated. No formal guidance on the thresholds for high or low quality guidelines is provided for AGREE II; however example approaches are discussed within the online instrument; with reference to these, we defined high quality guidelines as those where at least two domains scored ≥70%.

### Statement generation

Once the guidelines had been assessed and rated, any relevant recommendations from those guidelines rated as high quality were extracted to derive a set of statements regarding optimal management for the included topics (Additional file 2). All statements were checked for clinical content by a clinical fellow (a Speciality Registrar in NPN, who acted in an advisory role to the NGA technical team) and the committee chair. If no recommendations existed within the included guidelines for a particular review area, then no statement was produced.

### Formal consensus exercise

The formal consensus exercise was conducted over two committee meetings. Members of the guideline committee were individuals with expertise in the field of NPN, including lay members (mothers of babies who needed NPN). The following professions were included: neonatologist; neonatal nurse; paediatric dietitian; pharmacist; paediatrician; paediatric gastroenterologist; and paediatric surgeon. The meeting was facilitated by the NGA technical team which included systematic reviewers, a senior systematic reviewer and a guideline lead.

At the initial meeting the statements were presented to the committee, following an overview of the formal consensus process. All committee members took part in the formal consensus exercise, excluding the chair, as he had been involved in deriving the statements, and co-opted members (who are not routinely involved in developing recommendations). Committee members were asked to rate each statement based on their personal opinion of what they believed *‘best clinical practice’* would be*.* The statements were rated using a 9-point Likert scale, where 1 represents ‘strongly disagree’, 5 represents ‘neither agree nor disagree’, and 9 represents ‘strongly agree’. The participants were also able to state that they believed they had insufficient knowledge to provide a rating and there was also space for written comments about each statement. The questionnaire was completed anonymously with no prior discussion on the topic. Following the first round of consensus the NGA technical team calculated overall percentage agreement among committee members for each individual statement. The ratings were grouped into three categories: 1 to 3 (disagree), 4 to 6 (neither agree nor disagree), or 7 to 9 (agree). If a committee member indicated they had insufficient knowledge to provide a rating for a particular statement this was excluded from the calculation of agreement*.* Statements with 80% or greater agreement were kept, and used to inform recommendations. Statements with less than 60% agreement were discarded, unless there were obvious and addressable issues identified from any of the written comments which could be used to re-draft the statement. Any statements with 60–80% agreement were re-drafted, based on written comments. Re-drafted statements were placed into the same questionnaire format as round 1. Committee members were sent these revised statements electronically, and asked to rate them in the same way as in the first round, then to email back their completed forms. The same process of retaining or excluding statements was followed as in round 1; however no statements were redrafted in round 2 because they had already been redrafted after insufficient agreement in round 1, and further redrafting was not expected to lead to a higher level of agreement in a subsequent round of voting. During the second committee meeting, all the agreed statements were presented and discussed by the committee as a basis for them to generate their own guideline recommendations.

### Patient and public involvement

The protocol and the recommendations were developed by a committee which included lay members, who provided input throughout. There were two lay members on the committee, both of whom were mothers of babies who had required NPN at birth.

## Results

### Included guidelines

**Fig. 1:**
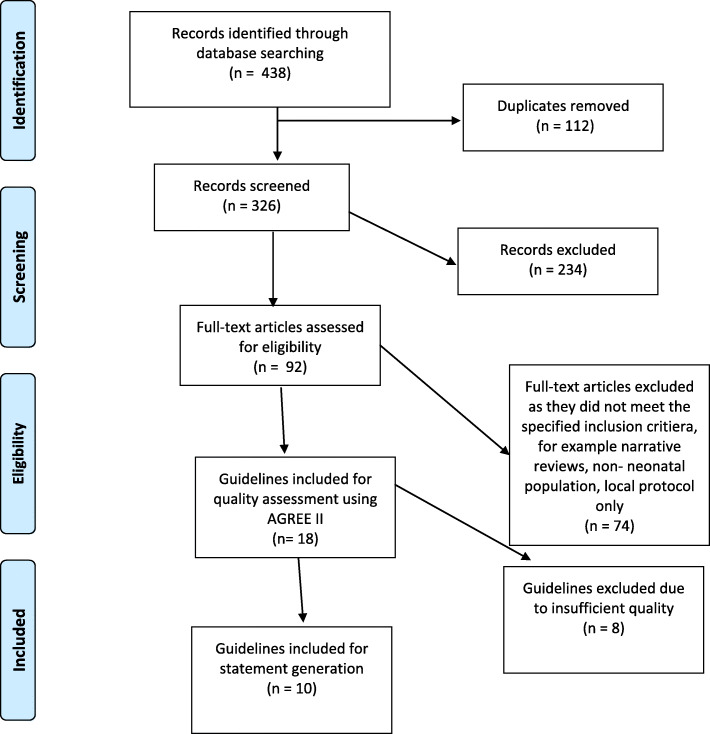
PRISMA flow chart of guideline selection for evidence review “General Principles” Neonatal parenteral nutrition NICE Guideline [NG154] 2020

A total of 325 records were identified by the search strategy, from which 18 full texts were included as potentially relevant guidelines for appraisal using AGREE II [18–35]. Ten guidelines were considered high quality and included [19, 22, 25, 26, 29, 31–35]. Seven of the guidelines were assessed as one, as they were developed using the same methods [25, 26, 31–35]. Details are presented in the PRISMA (preferred reporting items for systematic reviews and meta-analyses) flow diagram (Fig. 1). Guidelines were developed in Europe, China, Germany, and the United States.

### Quality of included guidelines

The included guidelines scored highly (≥ 70%) in at least two of the following four domains: scope and purpose, rigour of development, clarity of presentation, and editorial independence. Guidelines generally scored poorly (< 70%) in the following two domains: stakeholder involvement and applicability; guidelines were not developed by appropriate stakeholders, or did not demonstrate that the views of intended users were represented.

### Formal consensus; round 1

We conduced round 1 as a face to face exercise, as part of the committee meeting. The formal consensus exercise had previously been discussed with the committee at an earlier meeting to explain the process, and gain their views on taking part. Prior to start of the formal consensus exercise, the methods were re-explained, following on from which, the statements were provided to the committee members, and these were then individually and anonymously rated.

Twenty-eight statements were drafted using the included guidelines and presented in round 1 of the formal consensus exercise. The draft statements covered all of the specified topic areas: overall level of included vitamins (*n* = 7), general practice for fluid volume (*n* = 2), overall levels of blood and urinary electrolytes (*n* = 4), overall level of included minerals (n = 4), overall level of included trace elements (n = 7), delivery of lipids via syringe or bags (*n* = 1), and filtration and protection from light (*n* = 3). Ten of these statements reached ≥80% agreement in round 1. Nine statements had < 60% of agreement and were discarded. Six statements had between 60 and > 80% and these were redrafted for rating by the committee in round 2. A further three statements that had < 60% agreement demonstrated obvious and addressable issues; these statements were redrafted for assessment by the committee in round 2. (For example, a statement on multivitamins received a number of comments, saying it was too broad; therefore this was de-drafted to give additional detail).

### Formal consensus; round 2

Due to time restraints during the committee meeting, round 2 was conducted remotely with all individuals being emailed the redrafted statements. Each committee member completed their ratings of the re-drafted statements and then returned their questionnaires. The reviewers then assessed agreement of all returned questionnaires. In this second round, seven of the nine statements reached ≥80% agreement and were included for the discussion with the committee. The remaining two statements did not reach sufficient agreement and were discarded. After the two rounds of formal consensus the committee had reached agreement on 17 statements.

Despite the statements being developed from guidelines deemed high quality, some of the statements did not reflect an accurate representation of the committee’s opinion of best practice, or the statements were considered ambiguous and therefore not helpful for developing guidelines. Examples of rejected statements are presented in the supplementary material (Additional file 2).

### Generation of recommendations

For each specified topic area the agreed statements were re-presented to the committee and discussed. This was considered the starting point to generate the recommendations which would appear in the guideline. Even though the statements were agreed upon by the committee, these were statements and not recommendations in themselves. The statements were not necessarily worded in the way that the committee wanted, or lacked detail or clarity. Many of the recommendations made within NICE guidelines are highly complex, and need detail to ensure clarity and understanding, potentially across a range of situations. Therefore the agreed statements were modified using the committee’s experience and expertise. The committee members all discussed and agreed the wording, and the final recommendations can be seen in the published guideline [16].

### Lessons learnt

This case study has shown that using formal consensus during guideline development can improve transparency in generating recommendations. However, some important considerations should be taken into account following this case study. We developed statements based on previously published guidelines, as it was agreed the statements would then be based on the highest quality published evidence available. However if no guidelines are in existence alternative methods of generating statements may need to be considered. Additionally despite published guidelines being considered the best available evidence, their use risked simply re-iterating recommendations which had potentially been developed using informal consensus themselves. A way to help combat this in future would be to only include guidelines which scored highly in the domain “rigour of development” as assessed by the AGREE II instrument [17]. It is also important to consider who is involved in statement generation, or checking statements for clinical accuracy. Our statements were extracted from the published guidelines and then checked by a clinical fellow and the committee chair; it is important that this is undertaken by at least two individuals to reduce any risk of personal bias. When drafting the statements it is important to ensure the extracted information answers the question which needs addressing. In this case study we included one statement on filters used for NPN, and this statement reached agreement; however, the statement was not reflective of the real issue which the committee wished to discuss. Therefore, despite reaching agreement, it was not helpful when developing the eventual recommendations; when drafting statements, the question under review should remain at the forefront for inclusion.

It became clear that early discussion with the committee was important for uptake and acceptance of formal consensus. Using any non-standard methodology can be met with concern, especially by those who have never used the process before. Explaining the process and the reasons behind the decision to use nominal group technique are important to ensure all members are confident in carrying out the exercise. Discussing the use of formal consensus rather than a standard evidence review should be a shared decision between the technical team and the committee, to ensure all individuals are confident in the rigour and relevance of the methodology. Within a guideline committee the chair is a vital member who provides support to other committee members; a potential consideration for improved uptake and acceptance of formal consensus methods could be training the chair in these methods.

It may be necessary to conduct a standard systematic review to demonstrate no current evidence is available to give confidence to the committee that formal consensus is required. This also supports the importance of selecting the appropriate topic for formal consensus; if published evidence exists then a standard systematic review should be conducted to provide the committee with data to consider prior to making recommendations. However, topics which have limited evidence but are contentious should be considered, as formal consensus provides a transparent way to deal with potentially conflicting views [7].

A further consideration for the methodological process is the use of arbitrary thresholds in selecting which statements are included after voting. We used the standard, commonly used cut-offs; however, if different thresholds were used the final included set of statements may have been different and potentially this is an area for future research to determine if the chosen thresholds have any significant impact on overall outcomes.

Overall the use of formal consensus worked efficiently within this case study and the results support previous evidence which suggests formal consensus enhances the decision making process, and adds a level of quality assurance to the process [36]. However, these methods may not be suitable for all topics, determining criteria for when to use these methods should be an ongoing process. We suggest topics with no, or limited published evidence, and those topics where controversy in the evidence exists as an initial consideration to use formal consensus methods to help develop recommendations within guidelines. Additionally, formal consensus methodology may be helpful in developing clinical management strategies for COVID-19 until sufficient evidence becomes available to support more formal systematic reviews.

We have provided a case study, demonstrating that formal consensus can successfully be implemented within guideline development; however, this case study does not evaluate the process from the committee members point of view. Participants expectation and experiences of using formal group technique can be found in a further publication by Roberts et al. 2019 [37].

## Discussion

### Principal finding

Formal consensus methods can be usefully implemented during guideline development for generation of recommendations. The method provides all members equal input to the topics deemed necessary for recommendations.

### Implications and interpretation

Guidelines are based on the best available evidence, and across healthcare agencies, such as NICE, [1] WHO, [11] and PBAC [12] these guidelines are developed with a focus on conducting systematic reviews, anticipating sufficient evidence is available to collate data. These processes are deemed appropriate as health services acknowledge the importance of evidence based care. When limited evidence is available during guideline development, recommendations still need to be derived, as Eccles et al. state, *“Limiting recommendations to areas without evidence would reduce the scope of the guideline*” [38]. In general, where evidence is lacking, recommendations are made through informal consensus. However, this study has shown that using formal consensus methods during guideline development can improve transparency. The methodology allows stakeholders to clearly trace back how decisions have been made, giving support to these methods as compared to informal discussion. A previous review on consensus methods stated that a “danger with group consensus occurs when there is no rigour in the methods used”, [15] we believe the methods we outline in this report demonstrate clear and transparent group consensus is possible, and should be encouraged. Our results consolidate the findings of previous research which demonstrate formal consensus can be used to derive recommendations; for example, the nominal group technique was used to develop a primary care guideline for dementia [39].

Formal consensus methods are not new, and other studies have been conducted which demonstrate their use within guideline development, aiming to provide practical guidance on implementation [40]. However, uptake is still not widespread, we are confident our case study provides a helpful demonstration of how these methods can be used simply within a guideline setting. Furthermore the methods are supported by past research which suggests a formal consensus method which combines nominal group technique with the Delphi approach can result in greater understanding across groups, and greater reliability [41].

### Strengths and limitations

Despite this being a single case study to test formal consensus methods within a NICE guideline committee, the study had numerus strengths: The process was conducted rigorously, with systematic and transparent methods throughout, two reviewers conducted the search and quality assessment to reduce bias, and generated statements were checked by a clinical fellow and the committee chair to ensure accurate clinical context. Additionally, only high quality guidelines were included to develop the statements. When considering the final published guideline resulting from this review, it is important to state that it would be considered as high standard according to the AGREE II tool, and this would remain true even if informal consensus methods had not been used. However, the use of formal consensus methods where no published evidence exists, adds additional value to the “rigour in development” domain, due to the transparency of the process as compared to informal consensus.

One consideration when using the formal consenting procedure over a standard evidence review during guideline development is the time taken during the committee meeting. After the initial voting the technical team are required to calculate agreements and then a second round of voting is carried out. In this study the second round was conducted remotely as there was not sufficient time during the meeting; however, this could be overcome if the formal consensus exercise was planned in advance to be conducted during a two day meeting, allowing the two rounds to be split over the two days. This would ensure the same number of people complete both rounds of voting; in this study all committee members completed the remote voting; however, there is a risk with remote voting that people do not send their responses back.

It should also be acknowledged that following the agreement of statements, the committee still used informal consensus to generate their final recommendations, and as such the bias of informal group decision making cannot be excluded entirely. It is widely accepted that guideline development cannot be based on evidence alone, there is always some discussion, [42] using and documenting formal consensus makes this process more explicit and transparent. Additionally the process provides an opportunity for committee members to write comments anonymously, thus those less dominant characters can document their viewpoint, ensuring their views are discussed, this helps to limit bias and ensure all points of view are included. An interesting area for future research would be to investigate the potential impact that different committee members may have on the overall recommendations produced. However, we are confident the recommendations would not be significantly different, NICE guidelines are externally validated through a stakeholder consultation process, this procedure safeguards against any potential bias brought by individual committee members.

It is important to acknowledge that in guideline development, using formal consensus methods is relevant even when a standard evidence review is conducted. Following presentation of evidence from systematic reviews, there is always discussion within the committee, and the use of formal consensus methods may be considered as a complementary procedure.

## Conclusion

With appropriate planning and selection of topics, formal consensus should be considered for use within guideline development when agreed criteria are met. The process provides transparent methodology, ensuring stakeholders can see how recommendations were developed, despite limited evidence on a topic being available.

## Supplementary Information

**Additional file 1.** Search strategy: The search strategy used for the evidence review “General Principles” Neonatal parenteral nutrition within the NICE Guideline [NG154] 2020.

**Additional file 2.** Statements: Tables to show the generated statements for both round 1 and round 2 of consensus, plus example excluded statements. Used for the evidence review “General Principles” Neonatal parenteral nutrition NICE Guideline [NG154] 2020.

## Data Availability

Data sharing is not applicable to this article as no datasets were generated or analysed during the current study. For further information, please contact the corresponding author: patrice.carter@ucl.ac.uk.

## Abbreviations

AGREE II: Appraisal of Guidelines for Research and Evaluation
NGA: National Guideline Alliance
NICE: National Institute for Health and Care Excellence
PRISMA: Preferred Reporting Items for Systematic Reviews and Meta-Analyses
NPN: Neonatal Parenteral Nutrition
