# Supplementary material for: A demonstration of using formal consensus methods within guideline development; a case study
Source: BMC Med Res Methodol. 2021 Apr 17;21:73. doi: 10.1186/s12874-021-01267-0 (PMC8052943; doi:10.1186/s12874-021-01267-0)
Supplement: Supplementary file 1 — Additional file 1. Search strategy: The search strategy used for the evidence review “General Principles” Neonatal parenteral nutrition within the NICE Guideline [NG154] 2020. [file 12874_2021_1267_MOESM1_ESM.docx]

Additional file1. Search Strategy for evidence review “General Principles” Neonatal parenteral nutrition NICE Guideline [NG154] 2020

Medline; Medline EPub Ahead of Print; and Medline In-Process & Other Non-Indexed Citations

| **#** | **Searches** |
| --- | --- |
| 1 | INFANT, NEWBORN/ |
| 2 | (neonat$ or newborn$ or new-born$ or baby or babies).ti,ab. |
| 3 | PREMATURE BIRTH/ |
| 4 | ((preterm$ or pre-term$ or prematur$ or pre-matur$) adj5 (birth? or born)).ab,ti. |
| 5 | exp INFANT, PREMATURE/ |
| 6 | ((preterm$ or pre-term$ or prematur$ or pre-matur$) adj5 infan$).ti,ab. |
| 7 | (pre#mie? or premie or premies).ti,ab. |
| 8 | exp INFANT, LOW BIRTH WEIGHT/ |
| 9 | (low adj3 birth adj3 weigh$ adj5 infan$).ti,ab. |
| 10 | ((LBW or VLBW) adj5 infan$).ti,ab. |
| 11 | INTENSIVE CARE, NEONATAL/ |
| 12 | INTENSIVE CARE UNITS, NEONATAL/ |
| 13 | NICU?.ti,ab. |
| 14 | or/1-13 |
| 15 | exp CHILD/ |
| 16 | child$.ti,ab. |
| 17 | exp INFANT/ |
| 18 | infan$.ti,ab. |
| 19 | exp PEDIATRICS/ |
| 20 | p?ediatric$.ti,ab. |
| 21 | or/15-20 |
| 22 | PARENTERAL NUTRITION/ |
| 23 | PARENTERAL NUTRITION, TOTAL/ |
| 24 | PARENTERAL NUTRITION SOLUTIONS/ |
| 25 | ADMINISTRATION, INTRAVENOUS/ and (nutrition$ or feed$ or fed$).ti,ab. |
| 26 | INFUSIONS, INTRAVENOUS/ and (nutrition$ or feed$ or fed$).ti,ab. |
| 27 | CATHETERIZATION, CENTRAL VENOUS/ and (nutrition$ or feed$ or fed$).ti,ab. |
| 28 | exp CATHETERIZATION, PERIPHERAL/ and (nutrition$ or feed$ or fed$).ti,ab. |
| 29 | ((parenteral$ or intravenous$ or intra-venous$ or IV or venous$ or infusion?) adj3 (nutrition$ or feed$ or fed$)).ti,ab. |
| 30 | ((peripheral$ or central$) adj3 line? adj3 (nutrition$ or feed$ or fed$)).ti,ab. |
| 31 | (catheter$ adj3 (nutrition$ or feed$ or fed$)).ti,ab. |
| 32 | (drip? adj3 (nutrition$ or feed$ or fed$)).ti,ab. |
| 33 | or/22-32 |
| 34 | exp GUIDELINE/ |
| 35 | guideline.pt. |
| 36 | guideline?.ti. |
| 37 | or/34-36 |
| 38 | (standard$ adj3 protocol?).ti. |
| 39 | "European Society of Paediatric Gastroenterology, Hepatology and Nutrition".ti,ab. |
| 40 | ESPGHAN.ti,ab. |
| 41 | "European Society for Clinical Nutrition and Metabolism".ti,ab. |
| 42 | ESPEN.ti,ab. |
| 43 | "European Society of Paediatric Research".ti,ab. |
| 44 | ESPR.ti,ab. |
| 45 | "American Society for Parenteral and Enteral Nutrition".ti,ab. |
| 46 | ASPEN.ti,ab. |
| 47 | "British Association for Parenteral and Enteral Nutrition".ti,ab. |
| 48 | BAPEN.ti,ab. |
| 49 | "The Parenteral and Enteral Nutrition Group of the British Dietetic Association".ti,ab. |
| 50 | PENG.ti,ab. |
| 51 | "The British Society of Paediatric Gastroenterology, Hepatology and Nutrition".ti,ab. |
| 52 | BSPGHAN.ti,ab. |
| 53 | "National Nurses Nutrition Group".ti,ab. |
| 54 | NNNG.ti,ab. |
| 55 | "Patients on Intravenous and Nasogastric Nutrition Therapy".ti,ab. |
| 56 | PINNT.ti,ab. |
| 57 | "British Pharmaceutical Nutrition Group".ti,ab. |
| 58 | BPNG.ti,ab. |
| 59 | "British Association of Perinatal Medicine".ti,ab. |
| 60 | BAPM.ti,ab. |
| 61 | or/39-60 |
| 62 | (14 or 21) and 33 and 37 |
| 63 | (14 or 21) and 33 and 38 |
| 64 | (14 or 21) and 33 and 61 |
| 65 | or/62-64 |
| 66 | limit 65 to english language |
