# Supplementary material for: A demonstration of using formal consensus methods within guideline development; a case study
Source: BMC Med Res Methodol. 2021 Apr 17;21:73. doi: 10.1186/s12874-021-01267-0 (PMC8052943; doi:10.1186/s12874-021-01267-0)
Supplement: Supplementary file 2 — Additional file 2. Statements: Tables to show the generated statements for both round 1 and round 2 of consensus, plus example excluded statements. Used for the evidence review “General Principles” Neonatal parenteral nutrition NICE Guideline [NG154] 2020. [file 12874_2021_1267_MOESM2_ESM.zip › Additional file 2. consensus statements R3.docx]

Additional file 2: Generated Statements for for evidence review “General Principles” Neonatal parenteral nutrition NICE Guideline [NG154] 2020

**Table 1: Statements for Round 1**

| Name: | | | | | | | | | | |
| --- | --- | --- | --- | --- | --- | --- | --- | --- | --- | --- |
| **Overall level of included vitamins (lipid soluble and water soluble)** | | | | | | | | | | |
|  | **Strongly disagree** |  |  |  |  |  |  |  | **Strongly agree** | **Insufficient knowledge** |
| 1. Give parenteral vitamins to infants receiving parenteral nutrition. | 1 | 2 | 3 | 4 | 5 | 6 | 7 | 8 | 9 |  |
| Comments: | | | | | | | | | | |
| 1. Give parenteral vitamins daily to infants receiving parenteral nutrition. | 1 | 2 | 3 | 4 | 5 | 6 | 7 | 8 | 9 |  |
| Comments: | | | | | | | | | | |
| 1. Give parenteral vitamins as multivitamin products to infants receiving parenteral nutrition, where possible. | 1 | 2 | 3 | 4 | 5 | 6 | 7 | 8 | 9 |  |
| Comments: | | | | | | | | | | |
| 1. Parenteral vitamins may be given to infants receiving parenteral nutrition as multivitamin products or as individual vitamin products. | 1 | 2 | 3 | 4 | 5 | 6 | 7 | 8 | 9 |  |
| Comments: | | | | | | | | | | |
| 1. To improve vitamin stability, administer parenteral vitamin preparations together with the lipid emulsion or a mixture containing lipids. | 1 | 2 | 3 | 4 | 5 | 6 | 7 | 8 | 9 |  |
| Comments: | | | | | | | | | | |
| 1. Due to potential vitamin losses when given with a water-soluble solution, parenteral lipid soluble vitamins should be given with the lipid emulsion whenever possible. | 1 | 2 | 3 | 4 | 5 | 6 | 7 | 8 | 9 |  |
| Comments: | | | | | | | | | | |
| 1. Due to the hypothetical risk of adverse effects from transient high levels, intermittent substitution of parenteral vitamins is not recommended. | 1 | 2 | 3 | 4 | 5 | 6 | 7 | 8 | 9 |  |
| Comments: | | | | | | | | | | |
| **General practice for intravenous fluid volume** | | | | | | | | | | |
|  | **Strongly disagree** |  |  |  |  |  |  |  | **Strongly agree** | **Insufficient knowledge** |
| 1. Gradually increase fluid intake in infants after birth. | 1 | 2 | 3 | 4 | 5 | 6 | 7 | 8 | 9 |  |
| Comments: | | | | | | | | | | |
| 1. Gradually increase fluid intake in preterm and term neonates in the immediate postnatal phase. | 1 | 2 | 3 | 4 | 5 | 6 | 7 | 8 | 9 |  |
| Comments: | | | | | | | | | | |
| **Overall levels of blood and urinary electrolytes (Na, K, Cl)** | | | | | | | | | | |
|  | **Strongly disagree** |  |  |  |  |  |  |  | **Strongly agree** | **Insufficient knowledge** |
| 1. Due to the fluid intake replacing only the estimated losses in premature infants <1500g receiving parenteral nutrition, electrolyte supplementation may not be required in the first 5–7 days after birth. | 1 | 2 | 3 | 4 | 5 | 6 | 7 | 8 | 9 |  |
| Comments: | | | | | | | | | | |
| 1. Give electrolyte supplementation to infants receiving parenteral nutrition. | 1 | 2 | 3 | 4 | 5 | 6 | 7 | 8 | 9 |  |
| Comments: | | | | | | | | | | |
| 1. Begin electrolyte supplementation in infants receiving parenteral nutrition during the transition phase (contraction of extracellular fluid compartment/initial loss of body weight). | 1 | 2 | 3 | 4 | 5 | 6 | 7 | 8 | 9 |  |
| Comments: | | | | | | | | | | |
| 1. Maintain electrolyte homoeostasis while the infant is receiving parenteral nutrition. | 1 | 2 | 3 | 4 | 5 | 6 | 7 | 8 | 9 |  |
| Comments: | | | | | | | | | | |
| **Overall level of Magnesium** | | | | | | | | | | |
|  | **Strongly disagree** |  |  |  |  |  |  |  | **Strongly agree** | **Insufficient knowledge** |
| 1. Provide Magnesium to infants receiving parenteral nutrition. | 1 | 2 | 3 | 4 | 5 | 6 | 7 | 8 | 9 |  |
| Comments: | | | | | | | | | | |
| 1. Preterm infants on early parenteral nutrition during the first days of life may require lower amounts of magnesium, compared to growing stable preterm infants. | 1 | 2 | 3 | 4 | 5 | 6 | 7 | 8 | 9 |  |
| Comments: | | | | | | | | | | |
| 1. Adapt magnesium intakes to postnatal blood concentrations in infants receiving parenteral nutrition who have been exposed to maternal magnesium therapy. | 1 | 2 | 3 | 4 | 5 | 6 | 7 | 8 | 9 |  |
| Comments: | | | | | | | | | | |
| 1. Provide additional supplementation of magnesium for long term parenteral nutrition in infants when enteral nutrition provides 50% of the energy or less. | 1 | 2 | 3 | 4 | 5 | 6 | 7 | 8 | 9 |  |
| Comments: | | | | | | | | | | |
| **Overall level of trace elements (Zinc, Fluoride, Selenium, Copper, Chromium, Iodine, Manganese and Molybdenum)** | | | | | | | | | | |
|  | **Strongly disagree** |  |  |  |  |  |  |  | **Strongly agree** | **Insufficient knowledge** |
| 1. Give parenteral trace elements to infants receiving parenteral nutrition. | 1 | 2 | 3 | 4 | 5 | 6 | 7 | 8 | 9 |  |
| Comments: | | | | | | | | | | |
| 1. Give parenteral trace elements to infants receiving long-term parenteral nutrition. | 1 | 2 | 3 | 4 | 5 | 6 | 7 | 8 | 9 |  |
| Comments: | | | | | | | | | | |
| 1. Provide additional trace element supplementation to infants receiving long-term parenteral nutrition when enteral nutrition provides 50% of the energy or less. | 1 | 2 | 3 | 4 | 5 | 6 | 7 | 8 | 9 |  |
| Comments: | | | | | | | | | | |
| 1. Commence trace element supplementation in parenteral nutrition for premature infants <1500g when the infant begins gaining weight. | 1 | 2 | 3 | 4 | 5 | 6 | 7 | 8 | 9 |  |
| Comments: | | | | | | | | | | |
| 1. Commence trace element supplementation in parenteral nutrition for premature infants <1500g on day 5 of life. | 1 | 2 | 3 | 4 | 5 | 6 | 7 | 8 | 9 |  |
| Comments: | | | | | | | | | | |
| 1. Give parenteral trace elements as multi-trace element products for infants receiving parenteral nutrition, where possible. | 1 | 2 | 3 | 4 | 5 | 6 | 7 | 8 | 9 |  |
| Comments: | | | | | | | | | | |
| 1. Parenteral trace elements may be given to infants receiving parenteral nutrition as multi-trace element products or as individual trace element products. | 1 | 2 | 3 | 4 | 5 | 6 | 7 | 8 | 9 |  |
| Comments: | | | | | | | | | | |
| **Delivery of lipids via syringe or bags** | | | | | | | | | | |
|  | **Strongly disagree** |  |  |  |  |  |  |  | **Strongly agree** | **Insufficient knowledge** |
| 1. For parenteral solutions administered through a terminal filter, lipid emulsions (or all-in-one mixes) may be passed through a membrane pore size of 1.2-1.5 µm, and aqueous solutions may be passed through a 0.22 µm ﬁlter. | 1 | 2 | 3 | 4 | 5 | 6 | 7 | 8 | 9 |  |
| Comments: | | | | | | | | | | |
| **Filtration and protection from light** | | | | | | | | | | |
|  | **Strongly disagree** |  |  |  |  |  |  |  | **Strongly agree** | **Insufficient knowledge** |
| 1. Protect bags and administration sets for use in infant parenteral nutrition from light. | 1 | 2 | 3 | 4 | 5 | 6 | 7 | 8 | 9 |  |
| Comments: | | | | | | | | | | |
| 1. To prevent the generation of oxidants, parenteral solutions for premature infants should be protected against light. | 1 | 2 | 3 | 4 | 5 | 6 | 7 | 8 | 9 |  |
| Comments: | | | | | | | | | | |
| 1. Protect intravenous lipid emulsions for infant parenteral nutrition by validated light-protected tubing. | 1 | 2 | 3 | 4 | 5 | 6 | 7 | 8 | 9 |  |
| Comments: | | | | | | | | | | |

**Table 2: Example of reworded statements for round 2**

| Name: | | | | | | | | | | |
| --- | --- | --- | --- | --- | --- | --- | --- | --- | --- | --- |
| **Overall level of included vitamins (lipid soluble and water soluble)** | | | | | | | | | | |
|  | **Strongly disagree** |  |  |  |  |  |  |  | **Strongly agree** | **Insufficient knowledge** |
| 1. Give parenteral vitamins as multivitamin products to infants receiving parenteral nutrition. | 1 | 2 | 3 | 4 | 5 | 6 | 7 | 8 | 9 |  |
| Comments: | | | | | | | | | | |
| 1. To improve vitamin stability, administer parenteral vitamin preparations together with the lipid emulsion. | 1 | 2 | 3 | 4 | 5 | 6 | 7 | 8 | 9 |  |
| Comments: | | | | | | | | | | |
| 1. Parenteral lipid soluble vitamins should be given with the lipid emulsion whenever possible to remedy potential losses from vitamin oxidation. | 1 | 2 | 3 | 4 | 5 | 6 | 7 | 8 | 9 |  |
| Comments: | | | | | | | | | | |

**Table 3: Example of discarded statements**

|  |
| --- |
|  |
| Parenteral vitamins may be given to infants receiving parenteral nutrition as multivitamin products or as individual vitamin products |
| Due to the fluid intake replacing only the estimated losses in premature infants <1500g receiving parenteral nutrition, electrolyte supplementation may not be required in the first 5–7 days after birth. |
| Adapt magnesium intakes to postnatal blood concentrations in infants receiving parenteral nutrition who have been exposed to maternal magnesium therapy. |
